# Awareness, Attitudes and Self-Reported Practices Regarding Perinatal Mental Health and Depression Screening Among Healthcare Professionals in Serbia: An Exploratory Cross-Sectional Survey

**DOI:** 10.3390/jcm15218549

**Published:** 2026-11-03

**Authors:** Danilo Mladenović, Jelena Stojanov, Sanja Kostić, Nevena Mićanović, Marjana Gojković, Miloš Petronijević, Svetlana Vrzić Petronijević, Jovana Bjekić, Bojana Dunjić Kostić, Miloš Marković

**Affiliations:** 1 Special Hospital for Psychiatric Disorders Kovin, 26220 Kovin, Serbia; 2 Faculty of Medicine, University of Belgrade, 11000 Belgrade, Serbia; 3 Faculty of medicine, University of Niš, 18000 Niš, Serbia; 4 Center for Mental Health Protection, University Clinical Centre of Niš, 18000 Niš, Serbia; 5 Clinic for Gynecology and Obstetrics, University Clinical Centre of Serbia, 11000 Belgrade, Serbia; 6 Department for Gynecology, Health System “ Euromedik”, 11000 Belgrade, Serbia; 7 Clinic for Psychiatry, University Clinical Centre of Serbia, 11000 Belgrade, Serbia; 8 Human Neuroscience Group, Centre for Neuroscience and Neuromodulation, Institute for Medical Research, University of Belgrade, 11000 Belgrade, Serbia

**Keywords:** depression, postpartum, pregnancy, mass screening, health personnel, health knowledge, attitudes, practice, surveys and questionnaires

## Abstract

**Background:** Mood disorders, especially perinatal depression (PND), are a major public health concern, yet in Serbia there are no standardized screening protocols and limited data on healthcare professionals’ preparedness to recognize and manage it. **Aims:** To explore awareness, attitudes toward screening, self-reported familiarity, and self-reported clinical practices regarding PND among healthcare professionals in Serbia. **Method:** A cross-sectional, anonymous online survey was conducted in a convenience sample of 221 healthcare professionals (gynecology/obstetrics, pediatrics, psychiatry, midwifery, and community nursing) from two urban centers (Belgrade and Niš). A de novo 23-item survey was used. Group differences were tested with Fisher’s exact tests, and associations with demographic and professional characteristics were examined with penalized (Firth) logistic and ordinal regression models with psychiatry as reference category; *p*-values were Holm-adjusted within each family of tests. **Results:** Most respondents recognized pregnancy (85.5%) and the postpartum period (93.7%) as periods of increased risk for mental disorders and supported routine screening (91.0%). However, self-reported awareness of guidelines, screening timeframes, and diagnostic criteria was limited, particularly among non-psychiatric professionals. In multivariable analyses using psychiatry as the reference category, respondents working in gynecology/obstetrics had significantly lower odds of reporting awareness of national guidelines (OR = 0.120, 95% CI 0.038–0.327), international guidelines (OR = 0.176, 95% CI 0.044–0.575), recommended screening timeframes (OR = 0.053, 95% CI 0.007–0.237), and institutional clinical pathways (OR = 0.047, 95% CI 0.005–0.235). Respondents working in pediatrics also had lower odds of reporting awareness of national guidelines (OR = 0.121, 95% CI 0.043–0.297) and screening timeframes (OR = 0.032, 95% CI 0.005–0.132). Both non-psychiatric groups had higher odds of reporting poorer familiarity with ICD-10 diagnostic criteria and choosing referral to a psychiatrist rather than initiating screening themselves in the clinical vignette. All reported associations remained significant after Holm correction. **Conclusions:** In this convenience sample, gaps in self-reported familiarity, screening practices, and perceived institutional organization were evident, particularly outside psychiatry. Although the findings cannot be extrapolated to the national level without caution, they suggest that integrating routine screening into maternal care, targeted training, and updated national guidelines could improve the early detection and care of PND.

## 1. Introduction

Internationally, major professional bodies such as the American College of Obstetricians and Gynecologists (ACOG), the American Academy of Pediatrics (AAP), and the American Academy of Family Physicians (AAFP) recommend screening for depression at the initial prenatal visit, during pregnancy, and postpartum [1]. In the United Kingdom, the *Mothers and Babies: Reducing Risk through Audits and Confidential Enquiries across the UK* (MBRRACE-UK) report recommends that women are asked about any history of severe mental illness at their first contact with maternity services, and that those with such a history have a clearly documented management plan developed in collaboration with specialist perinatal mental health services [2]. In Serbia, in contrast, there are currently no nationally representative data on the prevalence of perinatal depression (PND), nor are there national screening protocols or comprehensive guidelines for healthcare professionals involved in the care of pregnant and postpartum women. A recent study on a Serbian sample of primiparous women (*n* = 188) reported depressive symptoms in 10.6% of women during the third trimester and in 29.3%, 26.6%, and 25.0% of women at 1, 6, and 12 months postpartum, respectively [3]. Internationally, the estimated incidence of postpartum depression is approximately 12%, while the reported prevalence is around 17% [4,5]. Prevalence is generally higher in low- and lower-middle-income countries than in high-income countries [6]; however, Serbia is classified as an upper-middle-income country [7], and these estimates therefore cannot be directly transferred to the Serbian context. Across settings, perinatal mental disorders remain substantially under-detected in routine care [8].

These data indicate a considerable public health issue, with challenges both for healthcare systems and for the healthcare professionals involved in the care of pregnant and postpartum women, i.e., gynecologists, pediatricians, psychiatrists, midwives, and nurses, who play an essential role in the early identification and management of perinatal mental health problems. In Serbia, perinatal psychiatry is not recognized as a subspecialty, and perinatal mental disorders are managed by general adult psychiatrists. There is no formally organized perinatal mental health service in the Serbian healthcare system, and the diagnosis and treatment of women with perinatal mental disorders depend on the organization and approaches of individual institutions.

Healthcare professionals’ preparedness to address PND is multidimensional. It comprises awareness of PND as a clinically relevant problem, knowledge of screening and diagnosis, attitudes toward screening, confidence in management, and actual clinical practice; these components are conceptually distinct and do not necessarily covary [9]. A systematic review of frontline professionals’ perinatal depression literacy showed that, although most professionals regard screening as important and recognize PND in case vignettes, their knowledge of screening methods is generally low [9]. Studies of midwives, community mental health providers, and obstetric and primary-care staff further report limited training in perinatal mental health [10], reluctance to initiate pharmacotherapy [11], and low confidence in interpreting screening results and initiating referrals [12]. At the system level, stigma, service fragmentation, and the absence of clear referral pathways have been identified as barriers to timely care [12,13,14]. Notably, most of this evidence comes from high-income countries [10,13,15] or from low- and lower-middle-income countries [16,17,18,19], and few studies have included psychiatrists alongside frontline providers. To the best of our knowledge, no study has examined Serbian healthcare providers’ awareness and self-reported practices related to perinatal depression or perinatal mental health in general. Such data are needed both to inform the prioritization and development of perinatal mental health services at the national level and to identify where professionals’ preparedness should be strengthened, in order to enable earlier intervention and reduce the risk of adverse outcomes, including maternal suicide, which in the UK and Ireland is the leading cause of direct maternal death between six weeks and one year after the end of pregnancy [20].

The primary aim of this exploratory study was to describe healthcare providers’ awareness, attitudes and self-reported clinical practices regarding PND in Serbia. The secondary aims were to describe perceived institutional organization and treatment-related confidence, and to compare these outcomes across professional groups, healthcare levels, and the two cities. Given the exploratory nature of the study, we formulated research questions rather than confirmatory hypotheses: (RQ1) To what extent are the healthcare professionals aware of the perinatal period as a period of increased risk for mental disorders and support routine screening? (RQ2) How familiar do they report being with national and international guidelines, screening timeframes, and ICD-10 criteria, and how do they report responding to women with possible perinatal mood symptoms? (RQ3) Are there any differences with regard to these between professional groups, healthcare levels, and cities? Based on previous findings that frontline providers often report limited training in perinatal mental health and rely on clinical judgment rather than routine screening [9,10,16,17], we expected psychiatry professionals to report greater familiarity and awareness than non-psychiatric professionals, and frontline providers in gynecology/obstetrics, pediatrics, midwifery, and community nursing to be familiar with routine screening practices and established referral pathways less often.

## 2. Materials and Methods

### 2.1. Research Design

This was an exploratory, cross-sectional, self-report survey study conducted among healthcare professionals involved in the care of women during the perinatal period. The study was conducted in two of the five statistical regions of Serbia defined by the Law on Regional Development [21], i.e., the Belgrade region and the Region of Southern and Eastern Serbia, specifically in the cities of Belgrade and Niš, the largest cities in the respective regions. Both public and private healthcare institutions at all levels of care (primary, secondary, and tertiary) were eligible.

### 2.2. Participants

Eligible participants were specialists and residents in gynecology/obstetrics, pediatrics, and psychiatry, as well as midwives (gynecology/obstetrics nurses/technicians) and visiting (community health) nurses working in Belgrade or Niš. Participants were recruited by convenience sampling; the electronic link to the survey was disseminated through professional networks and healthcare-related social media groups. Because the number of professionals reached through these channels could not be determined, the response rate could not be calculated. Professional groups were defined by combining the respondent’s field (survey item 3: gynecology/obstetrics, pediatrics, or psychiatry) with their professional role (item 5: specialist, resident, gynecology/obstetrics nurse/technician, or community health nurse/technician), which yielded the eight professional categories analyzed below.

### 2.3. Data Collection Tools

The survey was developed de novo, given the absence of previous research in Serbia and the context-specific nature of perinatal mental healthcare practices, professional roles, referral pathways, and institutional resources. No previously validated instrument covered these locally relevant aspects; the selection of content domains was informed by previous surveys of healthcare providers [9,16,18] and by current screening guidance [1,2].

Item generation proceeded in two stages. First, individual consultations were conducted with nine experienced healthcare professionals working with women in the perinatal period (three psychiatrists, four gynecologists/obstetricians and two midwives/nurses), from Belgrade and Niš. These professionals were not members of the author team and did not subsequently complete the survey. Consultations were semi-structured, and were documented through written notes; they were used to identify locally relevant practices, perceived knowledge gaps and barriers to care. Second, items were drafted against these themes and against the source documents above by the author team, which comprises psychiatrists, gynaecologists/obstetricians and a researcher with expertise in questionnaire design. An initial pool of 40 items was reduced to the final set by merging overlapping items and removing those judged not applicable to the Serbian context. The draft was reviewed internally by the coauthors for content relevance and clarity of wording. No formal expert review independent of the author team was undertaken, no cognitive interviewing was performed, and the instrument was not pilot-tested before distribution; these omissions are addressed in the Limitations section. The final survey comprised 23 items: two open numeric items (age, years of experience) and 21 yes/no or single-choice items. Yes/no items assessed respondents’ demographic and professional characteristics, perceptions of perinatal vulnerability to mental disorders, attitudes toward screening, familiarity with screening, diagnosis, and treatment, confidence in providing or referring for treatment, current screening practices, and perceived institutional organization. The complete survey is provided in Supplementary Files S1 and S2 in both the original Serbian and an English translation.

Items were grouped into six outcome domains, which are distinguished throughout the manuscript: (i) awareness, i.e., recognition of pregnancy and the postpartum period as periods of increased risk for mental disorders and of perinatal mental disorders as a public health issue (items 8, 9, and 11); (ii) attitudes toward screening (item 10); (iii) self-reported familiarity, i.e., the respondent’s perception of being sufficiently informed about national and international recommendations and screening timeframes, and familiarity with ICD-10 diagnostic criteria (items 12–15); (iv) self-reported clinical practice, i.e., the reported response to a clinical vignette and the reported frequency of discussing mental health and suggesting screening (items 16–18); (v) perceived institutional organization, i.e., the reported existence of a clinical pathway (item 19); and (vi) treatment-related confidence (items 20–23, psychiatry professionals only).

All domains were assessed by self-report; the survey therefore captures perceived familiarity and reported practice rather than objectively tested knowledge or observed clinical behavior. No composite or summed scores were computed at any point in the analysis.

Several items refer to peripartum “mental disorders” or “mood disorders” more broadly rather than to depression specifically, and the four treatment-confidence items refer to care during the perinatal period without specifying a condition. Results for these items are reported and interpreted according to the construct each item assesses, and depression-specific conclusions are drawn only from items that explicitly refer to depression.

The clinical vignette (item 16) described symptoms such as anxiety, excessive worry, fear, panic, and irritability and asked whether the respondent would recommend mood disorder screening. The item therefore assessed the respondent’s reported willingness to recommend mood disorder screening in response to non-specific perinatal distress, rather than diagnostic accuracy for depression. These symptoms are not core diagnostic features of a depressive episode in ICD-10 or DSM-5, and the vignette was not intended to describe a prototypical depressive presentation. However, anxiety and depressive symptoms frequently co-occur during the perinatal period [22], and current guidelines recommend screening for both perinatal depression and anxiety [1]. Thus, the vignette presents symptoms that could reasonably prompt consideration of screening, without establishing a diagnosis of depression. Because the survey consists of single-item indicators of distinct content domains rather than a multi-item scale measuring a latent construct, internal consistency and factorial validity were not applicable, and the development procedure and item-level content mapping are documented in Supplementary Table S1 which maps each item to its content domain, the source guidance or prior survey it derives from, and the consultation conclusion it reflects; formal content validity was not established.

### 2.4. Procedure

The survey was administered online via Google Forms between 20 December 2024 and 17 March 2025, and responses were restricted to one submission per participant. Participation was voluntary and anonymous, and completion took approximately 5–7 min. Before accessing the survey items, participants were presented with information about the study and provided electronic informed consent. The study was approved by the Ethics Committee of the University Clinical Centre of Serbia (No. 307/21).

### 2.5. Data Analysis

Statistical analyses were performed using EZR (version 1.55). Because age and years of professional experience were not normally distributed, group comparisons for these variables were performed with the non-parametric Mann–Whitney U test. Associations between categorical variables were assessed with Fisher’s exact test; when exact computation was impractical because of the size of the contingency table, *p*-values were obtained by Monte Carlo simulation (100.000 replicates). Within-respondent comparisons of two binary items (e.g., perceived risk in pregnancy vs. the postpartum period) were assessed with the exact McNemar test, and within-respondent comparisons of confidence across treatment modalities were assessed with the Friedman test followed by pairwise Wilcoxon signed-rank tests.

Multiple comparisons were controlled with the Holm (step-down Holm–Bonferroni) procedure, applied separately within each family of tests: comparisons across the eight professional groups (Table 1; m = 10 tests), comparisons between psychiatry residents and specialists (Table 2; m = 4), comparisons across healthcare levels (Supplementary Table S2; m = 10), comparisons between the two cities (Supplementary Table S2; m = 10), pairwise comparisons across treatment modalities (m = 6). For the binary and ordinal regression models, Holm adjustment was applied to the nine predictor-coefficient tests within each model. For the multinomial regression model, adjustment was applied jointly to the 18 predictor-coefficient tests across the two non-reference outcome categories. Because the Holm procedure has no single fixed significance threshold, both raw and Holm-adjusted *p*-values are reported, and statistical significance was set at an adjusted *p* < 0.05.

For multivariable analyses, respondents were classified into three specialty areas according to their reported field of practice (item 3): psychiatry (*n* = 115), gynecology/obstetrics (*n* = 44), and pediatrics (*n* = 62), with psychiatry as the reference category. The prespecified predictors were sex, age, years of professional experience, city of employment, specialty area, and healthcare level.

Binary outcomes were analyzed using Firth’s penalized-likelihood logistic regression to reduce potential sparse-data bias and instability of maximum-likelihood estimates. Odds ratios (ORs) were reported with 95% profile penalized-likelihood confidence intervals, and statistical significance was assessed using penalized likelihood-ratio tests. Familiarity with ICD-10 diagnostic criteria (Question VIII) was analyzed using proportional-odds ordinal logistic regression, with response categories ordered from familiar and relevant, through familiar but not relevant, to unfamiliar. The proportional-odds assumption was evaluated using the Brant test. Responses to the clinical vignette were analyzed using multinomial logistic regression, with direct screening as the reference outcome category.

Multicollinearity was assessed using variance inflation factors (VIFs). Model calibration was additionally examined using the Hosmer–Lemeshow goodness-of-fit statistic, calculated from Firth-model predicted probabilities grouped into ten approximately equally sized groups. Internal validation of the six Firth-penalized logistic regression models was performed using 400 bootstrap resamples per model to estimate optimism-corrected discrimination (AUC), predictive accuracy (Brier score), and calibration slopes. The Hosmer–Lemeshow results were interpreted cautiously because this test is not specifically validated for Firth-penalized regression. Model fit was also summarized using Nagelkerke’s pseudo-R^2^, calculated from the unpenalized Bernoulli log-likelihood evaluated at the Firth estimates relative to an intercept-only reference model. As the primary aim of the regression analyses was to investigate associations rather than develop predictive models, measures of model fit, calibration, and predictive performance were considered supplementary to the interpretation of regression coefficients and their confidence intervals.

## 3. Results

A total of 221 healthcare professionals participated in the study, of whom 67.0% (*n* = 148) worked in Belgrade and 33.0% (*n* = 73) in Niš. The sample comprised 173 women (78.3%) and 48 men (21.7%). The mean age was 41.2 years, and the mean duration of professional experience was 13.6 years. Neither age nor professional experience differed significantly between women and men (Mann–Whitney U = 4479.5, *p* = 0.404 and U = 4488, *p* = 0.392, respectively). Respondents from Niš were significantly older than those from Belgrade (mean age 45.71 vs. 38.97 years; U = 3407, *p* < 0.001) and had longer professional experience (17.85 vs. 11.53 years; U = 3384, *p* < 0.001).

The sample included midwives (*n* = 24), visiting nurses (*n* = 21), pediatric residents (*n* = 16), psychiatry residents (*n* = 46), gynecology residents (*n* = 8), pediatric specialists (*n* = 24), psychiatry specialists (*n* = 69), and gynecology specialists (*n* = 13). Psychiatry professionals thus constituted 52.0% of the sample (115/221); because professional groups were unequally represented, group-specific rather than pooled percentages are emphasized below.

Differences in response distributions across the eight professional groups were assessed using Fisher’s exact test with Monte Carlo simulation. Both unadjusted and Holm-adjusted *p*-values, corrected for ten comparisons, are presented in Table 1. After Holm correction, statistically significant differences were observed in perceptions of pregnancy and the postpartum period as vulnerable periods, self-reported awareness of national and international guidelines, familiarity with recommended screening timeframes and ICD-10 diagnostic criteria, responses to the clinical vignette, and the reported availability of an institutional clinical pathway (all adjusted *p* < 0.05). No statistically significant differences were found in support for routine screening or in recognition of perinatal mental disorders as a public health issue.

Among psychiatry specialists (*n* = 69), a higher proportion identified the postpartum period (97.1%, *n* = 67) than pregnancy (78.3%, *n* = 54) as a period of increased vulnerability to mental disorders (exact McNemar test, *p* < 0.001). Psychiatry specialists were also more likely to report being sufficiently informed about national guidelines (59.4%, *n* = 41) than international guidelines (36.2%, *n* = 25; exact McNemar test, *p* < 0.001).

Among healthcare professionals other than psychiatry specialists and residents, no statistically significant between-group differences were observed in the reported frequency of discussing mental health (Fisher’s exact test, *p* = 0.09) or of suggesting screening for PND (Fisher’s exact test, *p* = 0.13). Most of these respondents reported that such discussions and recommendations would be guided by individual clinical judgment rather than routine practice.

Additional questions, administered exclusively to psychiatry residents and specialists, assessed self-reported confidence in providing or referring patients for four therapeutic interventions: pharmacotherapy, psychotherapy, non-invasive brain stimulation (NIBS), and electroconvulsive therapy (ECT). Response distributions and between-group comparisons are presented in Table 2. After Holm correction for four comparisons, a statistically significant difference between residents and specialists was observed only for NIBS (adjusted *p* = 0.037). Specialists more frequently reported feeling confident than residents (14.5% vs. 2.2%), whereas residents more frequently reported a lack of confidence (65.2% vs. 39.1%).

In within-group analyses, self-reported confidence differed significantly across the four treatment modalities among both residents (*n* = 46; Friedman χ^2^(3) = 72.88, *p* < 0.001) and specialists (*n* = 69; Friedman χ^2^(3) = 96.22, *p* < 0.001). Post hoc pairwise Wilcoxon signed-rank tests with Holm correction (m = 6 within each group) identified significant differences in five of six comparisons among residents (all adjusted *p* < 0.001), with no significant difference between NIBS and ECT (adjusted *p* = 0.248). Among specialists, all six pairwise comparisons were statistically significant (all adjusted *p* < 0.05), including NIBS versus ECT (adjusted *p* = 0.0038).

In both groups, the proportion reporting confidence was highest for psychotherapy (63.0% of residents and 69.6% of specialists), followed by pharmacotherapy (28.3% and 44.9%, respectively), NIBS (2.2% and 14.5%), and ECT (2.2% and 10.1%).

Comparisons across healthcare levels and between the two cities are presented in Supplementary Table S2. After Holm correction (m = 10 within each family), none of these comparisons reached statistical significance (all adjusted *p* ≥ 0.14). In within-respondent comparisons, healthcare professionals working at the tertiary level and respondents working in Belgrade were significantly more likely to identify the postpartum period than pregnancy as a period of increased vulnerability to mental disorders (exact McNemar test, *p* = 0.0074 and *p* < 0.001, respectively).

### Multivariable Regression Models

Multivariable regression analyses were conducted to examine associations between healthcare professionals’ demographic and professional characteristics and their self-reported awareness and clinical practices concerning perinatal depression. After adjustment for the prespecified covariates and application of the Holm–Bonferroni correction within each model, specialty area emerged as the most consistently associated factor. Statistically significant multivariable regression associations and model diagnostics are presented in Supplementary Table S3.

In the six Firth-penalized binary logistic regression models, respondents working in gynecology/obstetrics had significantly lower odds than those working in psychiatry of reporting sufficient awareness of national guidelines (OR = 0.120, 95% CI 0.038–0.327), international guidelines (OR = 0.176, 95% CI 0.044–0.575), and recommended screening timeframes (OR = 0.053, 95% CI 0.007–0.237). They also had lower odds of reporting the existence of an institutional clinical pathway for perinatal depression (OR = 0.047, 95% CI 0.005–0.235). Compared with psychiatry, pediatrics was additionally associated with lower odds of reporting sufficient awareness of national guidelines (OR = 0.121, 95% CI 0.043–0.297) and recommended screening timeframes (OR = 0.032, 95% CI 0.005–0.132). These associations remained statistically significant after Holm correction (all adjusted *p* < 0.05). The wide confidence intervals for some estimates indicate considerable uncertainty regarding the magnitude of the associations.

In the proportional-odds ordinal logistic regression model, both gynecology/obstetrics (OR = 31.76, 95% CI 11.06–91.26) and pediatrics (OR = 43.84, 95% CI 16.28–118.05) were associated with higher odds of reporting poorer familiarity with ICD-10 diagnostic criteria compared with psychiatry. Similarly, in the multinomial logistic regression model of responses to the clinical vignette, respondents working in gynecology/obstetrics (OR = 46.28, 95% CI 11.96–179.07) and pediatrics (OR = 20.13, 95% CI 7.20–56.25) had higher odds of choosing referral to a psychiatrist rather than initiating screening themselves, relative to respondents working in psychiatry. All four associations remained statistically significant after Holm correction (adjusted *p* < 0.001). Additionally, each additional year of professional experience was associated with higher odds of reporting awareness of international guidelines (OR = 1.17, 95% CI 1.05–1.31; adjusted *p* = 0.025). No other examined predictors showed statistically significant associations after correction for multiple comparisons.

Given the emphasis on examining associations rather than predicting individual outcomes, supplementary assessments of model fit, calibration, and predictive performance were conducted to evaluate model adequacy and identify potential limitations affecting the interpretation of the findings.

The six binary regression models demonstrated varying levels of model fit and predictive performance. Nagelkerke’s pseudo-R^2^ ranged from 0.095 to 0.367. Approximate Hosmer–Lemeshow assessments did not identify statistically significant discrepancies for five of the six Firth-penalized models; however, the model for recognition of perinatal mental disorders as a public health issue (Question IV) showed a significant discrepancy between predicted and observed outcomes (*p* = 0.005). Because the Hosmer–Lemeshow procedure is not specifically validated for Firth-penalized estimation, these findings were interpreted cautiously. Internal validation using 400 bootstrap resamples per binary model yielded optimism-corrected AUC values ranging from 0.583 to 0.804 and calibration slopes ranging from 0.605 to 0.863, indicating variable discrimination and suggesting some degree of overfitting.

Multicollinearity diagnostics revealed a strong correlation only between age and years of professional experience (Pearson’s r = 0.918), with variance inflation factors (VIFs) of 6.93 and 6.89, respectively. Both variables were retained in the prespecified primary models because they represent conceptually distinct demographic and professional characteristics. However, their mutually adjusted coefficients were interpreted cautiously because of the observed multicollinearity. To assess its potential influence on the main findings, sensitivity analyses were performed by refitting the six Firth-penalized logistic regression models after excluding age and years of professional experience separately. The direction of the associations between gynecology/obstetrics and psychiatry remained consistent across these alternative model specifications, although some odds ratios varied in magnitude.

The global Brant test did not indicate a statistically significant violation of the proportional-odds assumption for the ICD-10 model (χ^2^(9) = 12.33, *p* = 0.195).

## 4. Discussion

This study explored awareness, attitudes toward screening, self-reported familiarity, and self-reported clinical practices regarding perinatal mental health among healthcare professionals. Despite broad recognition of perinatal mental disorders as a public health issue, we observed gaps in self-reported familiarity with guidelines and screening, in reported screening practice, and in perceived institutional organization, particularly outside psychiatry. Because the sample was a convenience sample from two urban centers in which psychiatry professionals were over-represented, these findings describe the participating professional groups rather than providing estimates for Serbian healthcare professionals in general.

Although most respondents viewed pregnancy (85.5%) and the postpartum period (93.7%) as periods of increased risk for mental disorders, group-specific analyses revealed deviations from this perspective: a substantial proportion of psychiatry specialists (17.4%) considered pregnancy to be a protective factor against mental disorders, while 20.8% of midwives and 33.3% of visiting nurses believed that pregnancy does not differ in mental health vulnerability from other life periods. Furthermore, psychiatry specialists, respondents from Belgrade, and those working at the tertiary level of healthcare more often identified the postpartum period than pregnancy as a period of increased risk. A recent review [23] concludes that both pregnancy and the postpartum period are particularly vulnerable phases for the manifestation of maternal mental health problems, including depression. Additionally, studies conducted in Serbia demonstrate a high prevalence of depressive symptoms both during pregnancy and in the postpartum period [3], as well as an association between depressive symptoms in pregnancy and depressive symptoms postpartum [24].

On the other hand, the prevailing positive view regarding the need for screening for PND (91.0%), as well as the recognition of perinatal mental disorders as a public health issue (80.1%), is consistent with most previous research [9], although some studies, such as the one by Munsele et al., showed that 60% of respondents considered postpartum depression not common enough to warrant screening [19]. Notably, support for routine screening did not differ across professional groups. Positive attitudes toward screening thus appear to be present in all professional groups, which suggests that attitudinal barriers are unlikely to be the main obstacle to implementation in this setting.

A substantial proportion of respondents reported insufficient familiarity with timeframes for screening and uncertainty regarding diagnostic and treatment responsibility. These findings are consistent with the results reported by Branquinho et al., who indicated that in 8 out of 10 studies examining knowledge and the importance of screening, most participants demonstrated low levels of knowledge regarding screening methods [9]. Likewise, in a more recent study by Patabendige et al., less than half of medical professionals reported being familiar with the Edinburgh Postnatal Depression Scale (EPDS) [18]. Although they differed from the other groups in our study, a concerning proportion of psychiatry specialists reported being insufficiently informed about screening timeframes (63.8%), national guidelines (40.6%), and international guidelines (63.8%), while less than 50% of them considered ICD-10 diagnostic criteria to be relevant.

Regression analyses indicated that the most consistent correlates of survey outcomes were related to professional context rather than basic demographics. After adjustment, associations with sex, age, city of employment, and healthcare level generally did not remain statistically significant, suggesting that the observed variability is less related to demographic factors and more to differences in the type of clinical training and professional role. Specialty area, most notably psychiatry, showed the strongest and most consistent associations across items, particularly for outcomes reflecting self-reported familiarity with guidelines and diagnostic frameworks, reported screening-related decisions, and recognition of institutional pathways. These findings are in line with the expectation that clinicians with formal mental health training are more likely to be familiar with perinatal mental health guidance and structured assessment, whereas non-psychiatric professionals may rely more on ad hoc clinical judgment or referral-based strategies. Association between psychiatry and the vignette response should be explained, in Serbia there is option to refer someone to a more specialized professional based on clinical experience.

Our results indicate a dissonance between the perceived importance of perinatal mood symptoms and self-reported preparedness to initiate screening. When presented with vignette first line providers would refer a patient immediately to a psychiatrist rather than screen. Another finding of our study is the reported lack of established clinical pathways, particularly among pediatric and gynecology/obstetrics staff, which may represent a systemic problem; this is in line with a previous study by Pope et al., in which healthcare professionals also reported concerns that women might get lost in the system during referrals [15]. An important observation is that psychiatry specialists and residents, who reported the greatest familiarity with diagnostic frameworks, are not typically positioned as frontline providers for pregnant and postpartum women. Institutional ambiguity regarding roles and responsibilities reflects previously described systemic barriers, including fragmented care pathways and insufficient interdisciplinary coordination, which may delay the identification and treatment of PND [9,15].

Furthermore, non-psychiatric respondents reported that they would not routinely discuss mental health with their patients, but would rather do so only when their clinical impression suggested it. Because these data reflect self-reported rather than observed practice, they may, if anything, overestimate actual screening behavior. Gupta et al. also found that 89% of participants did not routinely screen patients in the perinatal period and that less than 50% had heard about the screening questionnaire [16]. Similarly, Nakidde et al. demonstrated that healthcare professionals often lack adequate education in this area, do not perform routine screening, and tend to rely on clinical judgment, most commonly referring patients to psychiatrists when necessary [17]. In a study by Nechaeva et al., midwives reported that they primarily focus on the physical health of their patients, often due to high workload [25].

Self-reported confidence differed across treatment modalities. Among psychiatry residents, confidence differed between pharmacotherapy and psychotherapy and between pharmacotherapy and NIBS or ECT, but not between NIBS and ECT. Psychiatry residents expressed the highest confidence in providing or referring patients to psychotherapy, whereas they felt least confident regarding NIBS and ECT. Specialists more often than residents reported feeling confident regarding NIBS, which was the only resident–specialist difference that remained significant after correction.

Regarding the treatment options, psychiatry specialists reported being most confident in recommending psychotherapy, which ACOG recommends as monotherapy only for mild to moderate depression [26], while fewer than 50% stated that they would feel confident prescribing pharmacotherapy. The lowest levels of confidence were observed for neuromodulation approaches, with only 14.5% of psychiatry specialists reporting confidence in recommending NIBS and 10.1% in recommending ECT. These findings are clinically relevant because current evidence-based guidelines indicate that several treatment modalities have a role in the management of PND, depending on illness severity, previous treatment response, maternal–fetal/infant risk–benefit assessment, and patient preferences. Cognitive–behavioral therapy is strongly recommended for mild to severe PND, antidepressant medication for severe depression during pregnancy, and ECT for therapy-resistant or life-threatening severe depression. Although evidence for NIBS remains more limited, it is considered a potential alternative for selected pregnant or postpartum women with mild-to-moderate depressive symptoms when available and clinically appropriate [27].

### 4.1. Implications for Healthcare Services, Policy, and Future Research

The findings of this study suggest a gap in the organization of perinatal mental healthcare in Serbia, as perceived by healthcare professionals. While most respondents recognized perinatal mental disorders as a public health issue, early identification was not reported to be consistently implemented in clinical practice. This discrepancy may reflect a broader mismatch between where women most frequently access care during the perinatal period and where mental health expertise is concentrated.

In Serbia, pregnant women primarily interact with gynecologists and midwives within antenatal services, while postpartum care is largely delivered through pediatric services and visiting nurses. Psychiatrists, although more familiar with diagnostic frameworks, screening practices, and treatment options, are typically involved only after referral. In our sample, self-reported familiarity with screening tools, guideline recommendations, and referral pathways was low outside psychiatry. In a system in which frontline providers are not sufficiently informed to identify perinatal mental health problems, a substantial proportion of women experiencing depressive symptoms may remain undetected and untreated. These results therefore could be a signal of a service-level issue rather than solely an individual awareness gap. If confirmed in healthcare system study with representative samples, this would imply that addressing PND in Serbia requires not only raising awareness among clinicians but also restructuring care pathways so that screening and initial identification occur within routine maternal and child health services.

From a policy perspective, our findings suggest that national guidance on depression could benefit from more explicit attention to the perinatal period, and that the integration of structured screening and referral pathways for PND into routine maternal and child health services merits further consideration. The current Serbian national guideline [28] acknowledges depression during pregnancy and the postpartum period but does not specify the timeframes for screening or provide detailed diagnostic and treatment recommendations, which may contribute to the inconsistent identification of affected women and a reliance on individual clinical judgment. The introduction of structured national guidance for perinatal mental health could therefore be an important step in strengthening service delivery. In Serbia, the development and implementation of such guidance could involve collaboration between the Ministry of Health, national professional societies (such as psychiatric, gynecological, and pediatric associations), and public health institutions. A pragmatic approach would include concise clinical algorithms outlining when screening should occur, which tools should be used, and how referrals should be managed. Training initiatives should accompany these guidelines, particularly targeting non-psychiatric professionals who serve as the first point of contact for pregnant and postpartum women. Clarifying this distribution of responsibilities and establishing clear referral algorithms between gynecological, pediatric, and psychiatric services may reduce delays in identification, improve continuity of care, and reduce the uncertainty reported by many respondents in this study.

A feasible training model could focus on minimal yet practical competencies rather than extensive psychiatric training. For example, frontline providers could be trained to administer brief validated screening tools such as the EPDS, to recognize key warning signs, and to follow a structured referral pathway. Such targeted training could be incorporated into continuing medical education programs or professional development courses organized by medical chambers or professional associations. These interventions would likely require relatively modest resources, although their effect on early detection would need to be evaluated.

Furthermore, strengthening perinatal mental healthcare in Serbia may also require workforce-level reforms, as perinatal psychiatry is not yet systematically integrated into psychiatry training programs and fellowship-level training in this field is not currently available. Developing structured training pathways in perinatal mental health may therefore be an important step toward improving specialist expertise and expanding the capacity of the healthcare system to respond to maternal mental health needs. Although this study was conducted in two urban centers in Serbia, some of the challenges identified here may also be relevant to other middle-income health systems in which perinatal care is delivered mostly outside specialized perinatal mental health services; however, this would need to be examined directly.

The findings of this study also have implications for future research and clinical practice. Future studies should extend the present findings through larger, nationally representative samples that include healthcare professionals from different regions and levels of care, both rural and urban, through objective assessments of knowledge and observed practice, and through qualitative research exploring the barriers underlying limited screening and referral practices. Intervention studies are also needed to evaluate whether targeted training, the implementation of structured screening protocols, and clearly defined referral pathways improve healthcare professionals’ knowledge, confidence, and routine clinical practice. From a clinical perspective, the findings are consistent with integrating systematic screening for PND into routine maternal and child healthcare, particularly at the points of care where women most frequently interact with healthcare professionals.

### 4.2. Strengths and Limitations

The study offers insight into the awareness and self-reported clinical practices of healthcare professionals from three perspectives: the medical profession, the level of healthcare, and the city of employment. In contrast to many previous studies focusing on selected professional groups, often without psychiatrists, our study included a broad range of healthcare professionals, enabling interprofessional comparisons and improving the relevance of the findings within the national healthcare system [9,15,16,17,18,25]. To the best of our knowledge, it also provides the first data on this topic from Serbia.

Despite these strengths, the study has several limitations. First, participants were recruited by convenience sampling through professional networks and healthcare-related social media groups, which facilitated broad distribution but precluded tracking of the denominator; thus, the response rate and potential non-response bias could not be assessed. Professionals with a particular interest in perinatal mental health may have been more inclined to respond, and psychiatry professionals constituted more than half of the sample. Moreover, the study was conducted in only two of Serbia’s four university clinical centers, both located in large urban areas. Consequently, pooled percentages should not be interpreted as estimates for Serbian healthcare professionals in general, and the findings cannot be generalized to the national level or to rural settings. This limitation could be addressed in future research through structured, nationwide surveys conducted via official professional bodies, such as the Serbian Medical Chamber and other national professional associations.

Second, the survey was developed de novo. Although its content domains were informed by existing international and national guidance and consultations with experienced clinicians, as documented in Supplementary Table S1, no cognitive interviewing or pilot testing was performed, and no item-level or scale-level content validity index was computed. The survey was not designed as a multi-item psychometric scale, and its reliability and validity have not been formally established. Our findings should be read as self-reported awareness, familiarity and practice captured by a purpose-designed survey rather than as measurements with demonstrated reliability or objectively assessed knowledge, diagnostic accuracy, actual clinical behavior, or adherence to guidelines, and they may be affected by social desirability bias, which may lead to overestimation of favorable practices. The sample was predominantly female (78%), which limited the power to detect sex-related differences, and some response categories were sparse, which resulted in imprecise regression estimates despite penalized estimation. Finally, the cross-sectional design does not allow causal inferences about the factors associated with the outcomes. Nevertheless, the inclusion of professionals from multiple specialties, levels of healthcare (primary, secondary, tertiary, and private), and two major regional centers increased the heterogeneity of the sample, and the consistency of the observed group differences supports the robustness of the main pattern of results.

## 5. Conclusions

In this convenience sample of Serbian healthcare professionals, PND was widely recognized as a public health issue, yet substantial gaps were evident in self-reported guideline familiarity, screening practices, and perceived institutional care pathways, particularly outside psychiatry. The findings suggest a structural mismatch between the points of contact where women most frequently access perinatal care and the settings where mental health expertise is concentrated. Strengthening perinatal mental healthcare in Serbia may require system-level interventions, including targeted professional training, consideration of integration of screening into maternal and child healthcare services, clearer referral pathways between frontline providers and psychiatric services, and the development and updating of national guidelines. These implications should be confirmed in larger, representative samples and with objective measures of knowledge and practice; if confirmed, such approaches may offer a feasible strategy for improving the detection and management of perinatal mental health in Serbia and, potentially, in other health systems facing similar organizational challenges.

## Figures and Tables

**Table 1:** Questionnaire outcomes by professional group (number of respondents).

Item	Answer	Medical Professions (N)								*p* (Raw/Holm-Adjusted)
		MW	VN	CR-PED	CR-PSY	CR-GY	SP-PED	SP-PSY	SP-GY	
Pregnancy vulnerable period	sensitive	19	14	16	43	8	23	54	12	<0.001/0.002 *
	no difference	5	7	0	2	0	1	3	1	
	protective	0	0	0	1	0	0	12	0	
Postpartum vulnerable period	sensitive	20	14	16	45	8	24	67	13	<0.001/0.001 *
	no difference	4	6	0	1	0	0	0	0	
	protective	0	1	0	0	0	0	2	0	
Support for routine screening	yes	21	21	16	41	8	23	60	11	0.408/0.408
	no	3	0	0	5	0	1	9	2	
Public health issue	yes	17	14	16	39	8	22	50	11	0.032/0.064
	no	7	7	0	7	0	2	19	2	
Informed of national guidelines	yes	3	4	1	12	2	3	41	2	<0.001/<0.001 *
	no	21	17	15	34	6	21	28	11	
Informed of international guidelines	yes	1	1	3	5	3	4	25	0	<0.001/0.002 *
	no	23	20	13	41	5	20	44	13	
Familiar with screening timeframes	yes	1	0	0	12	0	2	25	1	<0.001/<0.001 *
	no	23	21	16	34	8	22	44	12	
Familiar with ICD-10 criteria	familiar-relevant	0	3	0	10	0	1	34	1	<0.001/<0.001 *
	familiar-irrelevant	4	5	0	18	2	3	31	1	
	unfamiliar	20	13	16	18	6	20	4	11	
After presented with vignette medical professional would	screen	2	6	1	26	2	6	50	0	<0.001/<0.001 *
	refer to psychiatrist	21	14	15	19	6	18	19	13	
	do nothing	1	1	0	1	0	0	0	0	
Institutional pathway exists	yes	1	5	0	6	1	4	20	0	0.014/0.039 *
	no	23	16	16	40	7	20	49	13	

MW—midwives, VN—visiting nurses, CR-PED—pediatric residents, CR-PSY—psychiatry residents, CR-GY—gynecology residents, SP-PED—pediatric specialists, SP-PSY—psychiatry specialists, SP-GY—gynecology specialists. *p*-values: Fisher’s exact test with Monte Carlo simulation, reported as raw/Holm-adjusted (m = 10); * adjusted *p* < 0.05.

**Table 2:** Self-reported confidence of psychiatry residents and specialists in providing or referring for therapeutic interventions.

Medical Professions	Answers			*p* (Raw/Holm-Adjusted)
	I Would Feel Confident	I Would First Need to Consult with a Colleague and Review the Professional Literature	I Would Not Feel Confident	
I feel confident in providing or referring a peripartum patient to psychotherapy, based on a risk-benefit assessment.				
clinical resident (psychiatrist)	29 (63.0%)	7 (15.2%)	10 (21.7%)	0.035/0.082
specialist (psychiatrist)	48 (69.6%)	17 (24.6%)	4 (5.8%)	
I feel confident in modifying or initiating psychiatric pharmacological treatment for a patient in the peripartum period, based on a risk-benefit assessment.				
clinical resident (psychiatrist)	13 (28.3%)	19 (41.3%)	14 (30.4%)	0.027/0.082
specialist (psychiatrist)	31 (44.9%)	30 (43.5%)	8 (11.6%)	
I feel confident in applying or referring a peripartum patient to non-invasive brain stimulation methods (e.g., rTMS, tES), based on a risk-benefit assessment.				
clinical resident (psychiatrist)	1 (2.2%)	15 (32.6%)	30 (65.2%)	0.009/0.037 *
specialist (psychiatrist)	10 (14.5%)	32 (46.4%)	27 (39.1%)	
I feel confident in applying or referring a peripartum patient to electroconvulsive therapy (ECT), based on a risk-benefit assessment.				
clinical resident (psychiatrist)	1 (2.2%)	11 (23.9%)	34 (73.9%)	0.168/0.168
specialist (psychiatrist)	7 (10.1%)	21 (30.4%)	41 (59.4%)	

Values are *n* (%). *p*-values: Fisher’s exact test, reported as raw/Holm-adjusted (m = 4); * adjusted *p* < 0.05.

## Data Availability

The raw data supporting the conclusions of this article will be made available by the authors on request.

## Supplementary Materials

The following supporting information can be downloaded at https://www.mdpi.com/article/10.3390/jcm15218549/s1: Table S1: Content mapping of survey items; Table S2: city of employment and healthcare level comparisons; Table S3: Statistically significant multivariable regression associations and model diagnostics; File S1: Survey on Healthcare Providers’ Attitudes Toward Women’s Mental Health in the Peripartum Period in English; File S2: Survey on Healthcare Providers’ Attitudes Toward Women’s Mental Health in the Peripartum Period in Serbian.
