# Supplementary material for: Awareness, Attitudes and Self-Reported Practices Regarding Perinatal Mental Health and Depression Screening Among Healthcare Professionals in Serbia: An Exploratory Cross-Sectional Survey
Source: J Clin Med. 2026 Nov 3;15(21):8549. doi: 10.3390/jcm15218549 (PMC13763777; doi:10.3390/jcm15218549)
Supplement: Supplementary file 1 [file jcm-15-08549-s001.zip › jcm-4608721-supplementary.pdf]

Table S1. Content mapping of survey items

Each item of the study-specific instrument is mapped to the content domain it was assigned to during instrument development, the source guidance or previously published provider survey that informed its content, and the conclusion from the consultation that it reflects. Reference numbers in parentheses refer to the manuscript reference list.

Item	Item content (abbreviated)	Content domain	Source guidance/prior survey informing the item	Consultation interview conclusions
Q1	Gender	Respondent characteristics	Standard respondent characteristic; used as a covariate in regression models	—
Q2	Age (open numeric)	Respondent characteristics	Standard respondent characteristic; used as a covariate	—
Q3	Clinical field (gynaecology/obstetrics, paediatrics, psychiatry)	Respondent characteristics	Defines the specialty-area variable used in analysis; professional groupings follow Branquinho et al. (9)	*These three fields are the groups in routine contact with women during the perinatal period in Serbia*
Q4	City of work (Belgrade, Niš)	Respondent characteristics	Study design: two of Serbia's four university clinical centres	—
Q5	Professional role (specialist, resident, clinical doctor, gynaecology–obstetrics nurse/midwife, community health nurse)	Respondent characteristics	Required to distinguish physicians from nursing and midwifery staff within each field	—
Q6	Level of institution (primary, secondary, tertiary, private)	Respondent characteristics	Levels of care at which perinatal contact occurs in the Serbian system	—
Q7	Years of professional experience (open numeric)	Respondent characteristics	Standard respondent characteristic; used as a covariate	—
Q8	Pregnancy as a period of altered vulnerability to mental disorders (protective / sensitive / no difference)	(i) Awareness	Howard & Khalifeh (8); Khamidullina et al. (23); response format after Nechaeva et al. (25)	*Clinicians hold divergent views on whether pregnancy itself raises or lowers risk*
Q9	Postpartum period as a period of altered vulnerability to mental disorders	(i) Awareness	Howard & Khalifeh (8); Khamidullina et al. (23); Zikic et al. (3) and Mladenović et al. (24) for the Serbian context	*The postpartum period was described as the more clearly recognised high-risk window*
Q10	Whether screening for mood disorders should be conducted routinely, and in which period	(ii) Attitudes toward screening	ACOG Clinical Practice Guideline No. 4 (1); MBRRACE-UK (2); Branquinho et al. (9); Munsele et al. (19)	*No routine screening in place; views divided between antenatal and postpartum screening*
Q11	Whether perinatal mental disorders constitute a significant public health issue	(i) Awareness	Howard & Khalifeh (8); Felker et al. (20); Branquinho et al. (9)	*Clinicians hold divergent views of perinatal mental disorders as a significant public health issue*
Q12	Whether sufficiently informed about current national recommendations	(iii) Self-reported familiarity	Serbian national clinical practice guideline for depression (28); Gupta et al. (16); Nakidde et al. (17)	*Limited awareness of the national guideline and of its perinatal content*
Q13	Whether sufficiently informed about current international recommendations on screening methods and treatment	(iii) Self-reported familiarity	ACOG Clinical Practice Guideline No. 4 (1); MBRRACE-UK (2); Radoš et al. (27); Patabendige et al. (18)	*Limited awareness of international guideline and of its perinatal content*
Q14	Whether sufficiently informed about the periods during which screening should be conducted	(iii) Self-reported familiarity	Screening timepoints specified in ACOG Clinical Practice Guideline No. 4 (1); Branquinho et al. (9); Gupta et al. (16)	*Uncertainty about when screening should occur was raised repeatedly*
Q15	Familiarity with, and perceived clinical relevance of, ICD-10 diagnostic criteria	(iii) Self-reported familiarity	ICD-10 categories F53 and F32–F33; Branquinho et al. (9)	*Criteria described as not reflecting presentations seen in practice*
Q16	Clinical vignette (non-specific anxiety, worry, fear, panic, irritability): would the respondent recommend mood disorder screening	(iv) Self-reported clinical practice	ACOG Clinical Practice Guideline No. 4 (1), which recommends screening for both perinatal depression and anxiety; vignette approach after Branquinho et al. (9)	*Non-specific anxiety and distress described as the typical presenting picture in frontline settings*
Q17	Reported frequency of discussing mental health at a perinatal check-up (non-psychiatrists)	(iv) Self-reported clinical practice	Nakidde et al. (17); Nechaeva et al. (25); Pope et al. (15)	*Discussion reported as ad hoc and driven by clinical impression rather than protocol*
Q18	Reported frequency of suggesting depression screening at a perinatal check-up (non-psychiatrists)	(iv) Self-reported clinical practice	Gupta et al. (16); Nakidde et al. (17)	*Unknown frequency of suggesting depression screening at a perinatal check-up among non-psychiatrists*
Q19	Whether the respondent's institution has a clearly established clinical pathway for screening, diagnosis and treatment	(v) Perceived institutional organisation	Webb et al. (12); Pope et al. (15)	*Absence of a defined pathway named as a principal obstacle to care*

Q20	Confidence in initiating or modifying psychiatric pharmacotherapy during the perinatal period (psychiatry only)	(vi) Treatment-related confidence	ACOG Clinical Practice Guideline No. 5 (26); Radoš et al. (27); Byatt et al. (11) on prescribing reluctance	*Risk–benefit uncertainty around prescribing in pregnancy and lactation*
Q21	Confidence in providing or referring for psychotherapy during the perinatal period (psychiatry only)	(vi) Treatment-related confidence	ACOG Clinical Practice Guideline No. 5 (26); Radoš et al. (27)	*Psychiatrists are likely confident in providing or referring for psychotherapy during the perinatal period*
Q22	Confidence in applying or referring for non-invasive brain stimulation (rTMS, tES) (psychiatry only)	(vi) Treatment-related confidence	Radoš et al. (27)	*Limited availability and limited familiarity with perinatal evidence*
Q23	Confidence in applying or referring for electroconvulsive therapy (psychiatry only)	(vi) Treatment-related confidence	Radoš et al. (27)	*Low confidence in applying or referring for electroconvulsive therapy among psychiatrists*

Abbreviations: ACOG, American College of Obstetricians and Gynecologists; ECT, electroconvulsive therapy; ICD-10, International Classification of Diseases, 10th revision; MBRRACE-UK, Mothers and Babies: Reducing Risk through Audits and Confidential Enquiries across the UK; rTMS, repetitive transcranial magnetic stimulation; tES, transcranial electrical stimulation.

Note: The consent statement preceding Q1 is not numbered as a survey item.

Table S2. Healthcare-level comparisons and city of employment.

Outcome/item	Primary (n=38)	Secondary (n=39)	Tertiary (n=123)	Private (n=21)	Raw p	Holm p
Pregnancy vulnerable period (yes)	37/38 (97.4%)	33/39 (84.6%)	103/123 (83.7%)	16/21 (76.2%)	0.192	0.962
Postpartum vulnerable period (yes)	38/38 (100.0%)	36/39 (92.3%)	114/123 (92.7%)	19/21 (90.5%)	0.380	1
Support for routine screening (yes)	37/38 (97.4%)	37/39 (94.9%)	110/123 (89.4%)	17/21 (81.0%)	0.144	0.865
Public health issue (yes)	35/38 (92.1%)	33/39 (84.6%)	95/123 (77.2%)	14/21 (66.7%)	0.072	0.633
National guidelines sufficiently informed	10/38 (26.3%)	9/39 (23.1%)	41/123 (33.3%)	8/21 (38.1%)	0.511	1
International guidelines sufficiently informed	7/38 (18.4%)	6/39 (15.4%)	23/123 (18.7%)	6/21 (28.6%)	0.662	1
Unfamiliar with screening timeframes	31/38 (81.6%)	32/39 (82.1%)	98/123 (79.7%)	19/21 (90.5%)	0.765	1
Unfamiliar with ICD-10 criteria	23/38 (60.5%)	18/39 (46.2%)	51/123 (41.5%)	16/21 (76.2%)	0.015	0.148
Vignette: would refer to psychiatrist (instead of screening or doing nothing)	24/38 (63.2%)	23/39 (59.0%)	62/123 (50.4%)	17/21 (81.0%)	0.077	0.572
Institutional pathway exists (yes)	7/38 (18.4%)	3/39 (7.7%)	26/123 (21.1%)	1/21 (4.8%)	0.108	0.651

Note. Raw p-values are those reported in the source table; Holm-adjusted p-values were calculated separately for the 10 healthcare-level comparisons (m = 10), using the displayed rounded raw p-values. Percentages use the respective group denominators.

Outcome/item	Belgrade (n=148)	Niš (n=73)	Raw p	Holm p
Pregnancy vulnerable period (yes)	129/148 (87.2%)	60/73 (82.2%)	0.036	0.358
Postpartum vulnerable period (yes)	142/148 (95.9%)	65/73 (89.0%)	0.076	0.688
Support for routine screening (yes)	133/148 (89.9%)	68/73 (93.2%)	0.619	1
Public health issue (yes)	117/148 (79.1%)	60/73 (82.2%)	0.721	1
National guidelines sufficiently informed	50/148 (33.8%)	18/73 (24.7%)	0.215	1
International guidelines sufficiently informed	33/148 (22.3%)	9/73 (12.3%)	0.100	0.902
Unfamiliar with screening time frames	119/148 (80.4%)	61/73 (83.6%)	0.713	1
Unfamiliar with ICD-10 criteria	73/148 (49.3%)	35/73 (47.9%)	0.202	1
Vignette: would refer to psychiatrist (instead of screening or doing nothing)	83/148 (56.1%)	43/73 (58.9%)	0.691	1
Institutional pathway exists (yes)	22/148 (14.9%)	15/73 (20.5%)	0.339	1

Note. Holm-adjusted p-values were calculated separately for the 10 city of employment comparisons (m = 10), using the displayed rounded raw p-values. Values are descriptive n (%).

Table S3. Statistically significant multivariable regression associations and model diagnostics.

Outcome	Significant predictor/contrast	OR (95% CI)	Raw p	Holm p	Pseudo-R²/HL p	Bootstrap corrected AUC/Brier/slope	Sensitivity OR full/age/experience

Support for routine screening	No previously significant coefficients	—	—	—	.133 / .423	.631 / .085 / .610	—
Public health issue	No previously significant coefficients	—	—	—	.095 / .005	.583 / .166 / .605	—
National guidelines sufficiently informed	Gynecology/obstetrics vs psychiatry	0.120 (0.038–0.327)	<.001	<.001	.280 / .383	.743 / .181 / .863	0.120 / 0.136 / 0.164
National guidelines sufficiently informed	Pediatrics vs psychiatry	0.121 (0.043–0.297)	<.001	<.001	same model	same model	—
International guidelines sufficiently informed	Gynecology/obstetrics vs psychiatry	0.176 (0.044–0.575)	0.003	0.025	.204 / .695	.702 / .144 / .775	0.176 / 0.237 / 0.289
International guidelines sufficiently informed	Experience (per year)	1.17 (1.05–1.31)	0.003	0.025	same model	same model	—
Unfamiliar with screening timeframes	Gynecology/obstetrics vs psychiatry	0.053 (0.007–0.237)	<.001	<.001	.367 / .967	.804 / .127 / .855	0.053 / 0.048 / 0.066
Unfamiliar with screening timeframes	Pediatrics vs psychiatry	0.032 (0.005–0.132)	<.001	<.001	same model	same model	—
Institutional pathway exists (yes)	Gynecology/obstetrics vs psychiatry	0.047 (0.005–0.235)	<.001	<.001	.218 / .190	.723 / .137 / .789	0.047 / 0.051 / 0.057
Unfamiliar with ICD-10 criteria	Gynecology/obstetrics vs psychiatry	31.76 (11.06–91.27)	<.001	<.001	Not applicable	—	—
Unfamiliar with ICD-10 criteria	Pediatrics vs psychiatry	43.84 (16.28–118.05)	<.001	<.001	Not applicable	—	—
Vignette: would refer to psychiatrist (instead of screening or doing nothing)	Gynecology/obstetrics vs psychiatry	46.30 (11.97–179.18)	<.001	<.001	Not applicable	—	—
Vignette: would refer to psychiatrist (instead of screening or doing nothing)	Pediatrics vs psychiatry	20.13 (7.20–56.25)	<.001	<.001	Not applicable	—	—

Note. Raw p-values for Firth-penalized binary logistic regression models (Questions III–VII and X) were obtained using penalized likelihood-ratio tests. For ordinal (VIII) and multinomial (IX) regression, raw p-values were obtained from their respective regression models. Holm p-values are adjusted within each model. The proportional-odds assumption of the ordinal logistic regression model was evaluated using the Brant test, which did not indicate a statistically significant global violation ($\chi^2(9) = 12.33$, $p = 0.195$).

File S1 - Survey in English

Survey on Healthcare Providers' Attitudes Toward Women's Mental Health in the Peripartum Period
This survey aims to address gaps in the availability of information and clinical practices in the field of women's mental health during the peripartum period.
Filling out the survey takes 5 to 7 minutes. Thank you for taking the time to contribute to a better understanding of the needs in the field of women's mental health.

1. **Gender:**

a. Female
b. Male

2. **Age (in years):_____________**
3. **Your field is:**

a. Gynecology/Obstetrics
b. Pediatrics
c. Psychiatry
4. **Place where you work:**
a. Niš
b. Belgrade
5. **You are:**
a. Specialist doctor
b. Resident
c. Clinical doctor – Medical Doctor
d. Gynecology-Obstetrics nurse/technician
e. Community health nurse/technician
6. **The institution you work in is:**
a. Primary healthcare level
b. Secondary healthcare level
c. Tertiary healthcare level
d. Private sector healthcare institution
7. **Years of experience: _____________**
8. **In your opinion and experience, the pregnancy period represents:**
a. A protective factor against the onset of mental disorders
b. A sensitive period with a higher risk of mental disorders
c. No difference compared to the period before pregnancy
9. **In your opinion and experience, the postpartum period represents:**
a. A protective factor against the occurrence of mental disorders
b. A sensitive period with a higher risk of mental disorders
c. No difference compared to the period before pregnancy
10. **Do you believe that mood disorder screening should be regularly conducted during the peripartum period?**
a. Yes, during the postpartum period
b. Yes, both during pregnancy and postpartum
c. No
11. **Do you consider mental disorders during the peripartum period to be a significant public health issue?**
a. Yes
b. No
12. **Are you sufficiently informed about the current recommendations in the Republic of Serbia regarding the treatment of peripartum mood disorders?**
a. Yes
b. No
13. **Are you sufficiently informed about current global recommendations concerning screening methods and the treatment of peripartum mood disorders?**
a. Yes
b. No
14. **Do you feel sufficiently informed about the periods during which screening for mood disorder symptoms should be conducted during the peripartum period?**
a. Yes
b. No
15. **Your opinion on the diagnostic criteria within ICD-10 for postpartum depression:**
a. I know the criteria and believe they are relevant for application in daily practice
b. I do not know the criteria
c. I know the criteria but believe they do not accurately reflect what I encounter in clinical practice
16. **If a patient in the peripartum period complains of symptoms such as: anxiety, excessive worry, fear, panic, irritability; would you recommend mood disorder screening?**
a. Yes
b. No
c. No, but I would suggest she consult a psychiatrist
d. Yes, but I am not sufficiently informed about which screening method to apply
17. **If a patient in the peripartum period comes for a check-up, how often would you talk to her about her mental health (this question applies to everyone except psychiatrists)?**
a. Never
b. Only if she complains
c. If I consider, based on clinical assessment, that it is necessary to discuss it
d. Almost always
e. I am a psychiatrist

18. **If a patient in the peripartum period comes for a check-up, how often would you suggest screening for peripartum depression (this question applies to everyone except psychiatrists)?**
a. Never
b. Only if she complains
c. If I consider, based on clinical assessment, that it is necessary to discuss it
d. Almost always
e. I am a psychiatrist

19. **Does the institution where you work have a clearly established clinical pathway for the screening, diagnosis, and treatment of peripartum depression?**
a. Yes
b. No

20. **(For psychiatrists only) I feel confident that, in the peripartum period, I can change or introduce psychiatric medication therapy for a patient, based on the assessment of benefits and risks:**
a. I would not feel confident
b. I would feel confident
c. I would need to primarily consult with a colleague and review professional literature
d. I am not a psychiatrist

21. **(For psychiatrists only) I feel confident that, in the peripartum period, I can provide or refer a patient for psychotherapy based on the assessment of benefits and risks:**
a. I would not feel confident
b. I would feel confident
c. I would need to primarily consult with a colleague and review professional literature
d. I am not a psychiatrist

22. **(For psychiatrists only) I feel confident that, in the peripartum period, I can apply or refer a patient for one of the non-invasive brain stimulation methods (rTMS, tES) based on the assessment of benefits and risks:**
a. I would not feel confident
b. I would feel confident
c. I would need to primarily consult with a colleague and review professional literature
d. I am not a psychiatrist

23. **(For psychiatrists only) I feel confident that, in the peripartum period, I can apply or refer a patient for electroconvulsive therapy based on the assessment of benefits and risks:**
a. I would not feel confident
b. I would feel confident
c. I would need to primarily consult with a colleague and review professional literature
d. I am not a psychiatrist

File S2 - Survey in Serbian

Упитник о ставовима здравствених радника о менталном здрављу жена у перипарталном периоду

Овај упитни има за циљ адресирање недостатака у нивоу доступности информација и клиничке праксе у области менталног здравља жена у перипарталном периоду.
Попуњавање анкете траје 5 до 7 минута. Хвала Вам што ћете издвојити то време као допринос бољем разумевању потреба у области менталногздравља жена.

1. Пол:
a. Женски
b. Мушки

2. Године старости (упишите број нпр. 55):___________________

3. Ваша област је:
a. гинекологија/акушерство
b. педијатрија
c. психијатрија

4. Место у коме радите:
a. Ниш
b. Београд

5. Ви сте
a. лекар специјалиста
b. лекар на специјализацији
c. клинички лекар-доктор медицине
d. гинеколошко-акушерска сестра/техничар
e. патронажна сестра/техничар

6. Установа у којој радите је:
a. примарног нивоа здравствене заштите
b. секундарног нивоа здравствене заштите
c. терцијарног нивоа здравствене заштите
d. здравствена установа у приватном сектору

7. Године искуства (упишите број нпр. 10):____________________

8. Период трудноће према Вашем мишљењу и искуству представља:
a. протективни фактор за појаву менталних поремећаја
b. осетљив период са већом ризиком од појаве менталних поремећаја
c. нема разлике у односу на период пре трудноће

9. Постпартални период према Вашем мишљењу и искуству представља:
a. протективни фактор за појаву менталних поремећаја
b. осетљив период са већом ризиком од појаве менталних поремећаја
c. нема разлике у односу на период пре трудноће

10. Да ли сматрате да би скрининг на поремећаје расположења требало редовно да се ради у перипарталном периоду:
a. Да, у постпарталном периоду
b. Да, и током трудноће и у постпарталном периоду
c. Не

11. Да ли сматрате да ментални поремећаји у перипарталном периоду представљају значајан јавно-здравствени проблем :
a. Да
b. Не

12. Да ли сте довољно информисани о важећим препорукама у Републици Србији (Национални водич добре клиничке праксе за дијагностиковање и лечење депресије) о лечењу перипарталних поремећаја расположења:
a. Да
b. Не

13. Да ли сте довољно информисани о важећим светским препорукама које се тичу метода скрининга и лечења перипарталних поремећаја расположења:
a. Да
b. Не

14. Да ли сматрате да сте довољно информисани о периодима у којима би требало да се обави скрининг на симптоматологију поремећаја расположења у перипарталном периоду:
a. Да
b. Не

15. Ваше мишљење о дијагностичким критеријумима у оквиру МКБ-10 за постпорођајну депресију:
a. Знам који су критеријуми и мислим да су релевантни за примену у свакодневној пракси
b. Не знам који су критеријуми
c. Знам који су критеријуми, али сматрам да не одсликавају добро оно што срећем у клиничкој пракси

16. Уколико би Вам се пацијенткиња која је у перипарталном периоду пожалила на симптоме као што су: анксиозност, претерана брига, страх, паника, раздражљивост; да ли бисте јој предложили скрининг на поремећаје расположења?
a. Да
b. Не
c. Не, али бих јој предложио/ла да се јави психијатру

d. Да, али нисам довољно информисан/а о који скрининг метод применити

17. Уколико Вам на преглед дође пацијенткиња у перипарталном периоду колико често ћете с њом разговарати о њеном психичком стању (питање је за све осим за психијатре):
a. Никада
b. Само уколико се она пожали
c. Уколико на основу клиничке процене сматрам да је потребно о томе разговарати
d. Скоро увек
e. Ја сам психијатар

18. Уколико Вам на преглед дође пацијенткиња у перипарталном периоду колико често ћете јој предложити скрининг на перипарталну депресију (питање је за све осим за психијатре) :
a. Никада
b. Само уколико се она пожали
c. Уколико на основу клиничке процене сматрам да је потребно о томе разговарати
d. Скоро увек
e. Ја сам психијатар

19. Да ли у установи у којој радите постоји јасно успостављен клинички пут за скрининг, дијагностику и лечење перипарталне депресије?
a. Да
b. Не

20. (Само за психијатре) Осећам се самоуверено да пацијенткињи у перипарталном периоду уз процену користи и ризика променим или уведем психијатријску медикаментозну терапију:
a. Не бих био самоуверен/а
b. Био/била бих самоуверен/а
c. Морао/ла бих да се првенствено консултујем са колегом или колегиницом и претражим стручну литературу
d. Нисам психијатар

21. (Само за психијатре) Осећам се самоуверено да пацијенткињи у перипарталном периоду уз процену користи и ризика пружим или упутим на психотерапију:
a. Не бих био самоуверен/а
b. Био/била бих самоуверен/а
c. Морао/ла бих да се првенствено консултујем са колегом или колегиницом и претражим стручну литературу
d. Нисам психијатар

22. (Само за психијатре) Осећам се самоуверено да пацијенткињи у перипарталном периоду уз процену користи и ризика применим или упутим на неку од метода неинвазивне мождане стимулације (rTMS, tES):
a. Не бих био самоуверен/а
b. Био/била бих самоуверен/а
c. Морао/ла бих да се првенствено консултујем са колегом или колегиницом и претражим стручну литературу
d. Нисам психијатар

23. (Само за психијатре) Осећам се самоуверено да пацијенткињи у перипарталном периоду уз процену користи и ризика применим или упутим на лечење електроконвулзивном терапијом:
a. Не бих био самоуверен/а
b. Био/била бих самоуверен/а
c. Морао/ла бих да се првенствено консултујем са колегом или колегиницом и претражим стручну литературу
d. Нисам психијатар
